# Fine-Scale Spatial Heterogeneity in the Distribution of Waterborne Protozoa in a Drinking Water Reservoir

**DOI:** 10.3390/ijerph120911910

**Published:** 2015-09-23

**Authors:** Jean-Baptiste Burnet, Leslie Ogorzaly, Christian Penny, Henry-Michel Cauchie

**Affiliations:** 1 Department of Environmental Research and Innovation (ERIN), Luxembourg Institute of Science and Technology, 41, rue du Brill, Belvaux L-4422, Luxembourg; E-Mails: jeanbaptiste.burnet@gmail.com (J.-B.B.); leslie.ogorzaly@list.lu (L.O.); christian.penny@list.lu (C.P.); 2 Department of Environmental Sciences and Management, Université de Liège (ULg), 165 avenue de Longwy, Arlon B-6700, Belgium

**Keywords:** *Giardia*, *Cryptosporidium*, spatial distribution, microbial risk monitoring, reservoir

## Abstract

Background: The occurrence of faecal pathogens in drinking water resources constitutes a threat to the supply of safe drinking water, even in industrialized nations. To efficiently assess and monitor the risk posed by these pathogens, sampling deserves careful design, based on preliminary knowledge on their distribution dynamics in water. For the protozoan pathogens *Cryptosporidium* and *Giardia*, only little is known about their spatial distribution within drinking water supplies, especially at fine scale. Methods: Two-dimensional distribution maps were generated by sampling cross-sections at meter resolution in two different zones of a drinking water reservoir. Samples were analysed for protozoan pathogens as well as for *E. coli*, turbidity and physico-chemical parameters. Results: Parasites displayed heterogeneous distribution patterns, as reflected by significant (oo)cyst density gradients along reservoir depth. Spatial correlations between parasites and *E. coli* were observed near the reservoir inlet but were absent in the downstream lacustrine zone. Measurements of surface and subsurface flow velocities suggest a role of local hydrodynamics on these spatial patterns. Conclusion: This fine-scale spatial study emphasizes the importance of sampling design (site, depth and position on the reservoir) for the acquisition of representative parasite data and for optimization of microbial risk assessment and monitoring. Such spatial information should prove useful to the modelling of pathogen transport dynamics in drinking water supplies.

## 1. Introduction

Man-made reservoirs are major drinking and recreational water resources, and nearly 7000 of them are used as drinking water supplies worldwide [1]. However, their microbial quality is threatened by a series of pathogenic microorganisms causing waterborne outbreaks of enteric diseases [2,3,4]. Over the past three decades, a significant number of cryptosporidiosis and giardiasis outbreaks have been associated with the consumption of contaminated drinking water drawn from lakes or reservoirs [5]. In 1993, during the Milwaukee cryptosporidiosis outbreak, *Cryptosporidium* was discharged into Lake Michigan following heavy rainfall and entered the drinking water treatment plant (DWTP). The treatment barrier did not prevent introduction of the pathogen into potable water, resulting in >400,000 estimated illness cases [6]. During the 2004 giardiasis outbreak in Bergen, Norway, *Giardia* cysts were also inadequately removed from untreated lake water by the DWTP, following rainfall-induced contamination of Lake Svartediket [7]. More recently in 2010, a cryptosporidiosis outbreak caused by *Cryptosporidium hominis* was reported in Östersund, Sweden, after suspected sewage contamination of Lake Storsjön from which drinking water was obtained. It is to date the largest waterborne outbreak (estimated 27,000 cases) recorded in Europe [8]. As a strategy to ensure safe drinking water supply, the World Health Organization recommends to identify and control potential microbial hazards from source to the tap [9]. This notably implies acquisition of preliminary knowledge on the origins, loads and distribution dynamics of pathogenic microorganisms in water. *Cryptosporidium* and *Giardia* are ubiquitous to surface water and are discharged by a variety of point and diffuse faecal pollution sources of human and animal origin [10]. Their distribution dynamics have been addressed over a range of temporal and spatial scales [11,12,13]. However, in lakes and reservoirs, spatial patterns have essentially been explored at the scale of the waterbody and samples most often collected from surface or near-surface layers [14,15]. One study examined the occurrence of *Cryptosporidium* oocysts at the bottom of a drinking water reservoir and measured higher oocyst densities than at its surface [16]. To our best knowledge, spatial distribution patterns within lakes and reservoirs have not been reported for *Cryptosporidium* oocysts and *Giardia* cysts at fine scale, although they have been addressed for other protozoans as well as for bacteria and viruses [17,18,19,20,21]. Importantly, because (oo)cysts are subject to both vertical and horizontal transport, surface sampling can potentially yield biased estimates of parasite load if variations within the water column are not known.

Absence of fine-scale spatial information is likely related to the inherent constraints associated with standardized quantitative detection of *Cryptosporidium* oocysts and *Giardia* cysts in aqueous matrices. Because of low background (oo)cyst levels in surface water, large volumes need to be concentrated for quantitative detection [22]. In addition, enumeration of (oo)cysts is time-consuming and expensive, thereby limiting the number of samples that can be collected and processed [23]. Despite these limitations, the standard enumeration of *Cryptosporidium* and *Giardia* has been performed in many countries to describe their occurrence in source water and to assess the associated microbial risk [14,24,25], since faecal indicators such as *Escherichia coli* cannot reliably predict that risk [16,26]. Given the fact that direct enumeration of *Cryptosporidium* and *Giardia* currently remains the best way to assess the associated microbial risk, sampling deserves careful design in order to take into account the extent of spatial and temporal distribution variability of both parasites in water. In a previous investigation, we have shown that *Cryptosporidium* and *Giardia* densities displayed substantial spatial and temporal variability at the scale of the rural Upper-Sûre catchment and its drinking water reservoir, which provides more than half of the drinking water demand of Luxembourg [27]. The aim of the present study was to focus on the distribution of the protozoan pathogens at high spatial resolution in the Upper-Sûre reservoir water column. Sampling was performed during the prevalence period of both parasites (December 2009 to March 2010), which is also the period of highest risk for the DWTP given high rainfall-induced runoff [27]. We aimed at obtaining “snapshots” of parasite spatial distribution by sampling entire cross-sections at meter-resolution in two reservoir zones of contrasting hydrodynamic behaviour. In order to understand the spatial patterns, surface and subsurface flow dynamics were also described and parasite distribution compared with those of *E. coli* and other water quality parameters. 

## 2. Materials and Methods

### 2.1. Site Selection and Sampling

**Figure 1:**
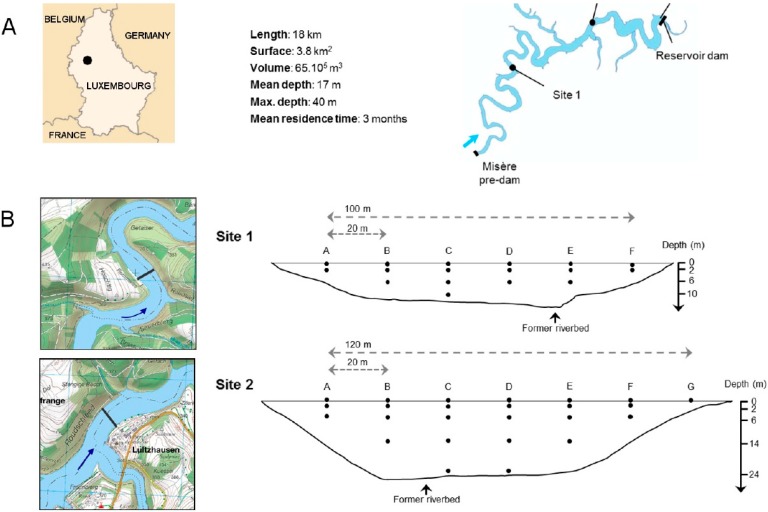
(**A**) Map of the Upper-Sûre reservoir and location of site 1 and site 2. The DWTP inlet is located 5 km downstream site 2; (**B**) (left) Close-up of site 1 and site 2. The dashed lines illustrate the former riverbed, while the thick black lines indicate the position of each cross-section. Flow direction is given by the blue arrow, (right) Sampling grid for cross-sections 1 and 2.

Two sites were selected in the Upper-Sûre reservoir, which is located in a rural 428 km^2^-large watershed in the North of Luxembourg (Figure 1A). The reservoir is the main water supply of the country, delivering more than 50% of the total drinking water volume and reaching nearly 80% of the population. As a typical riverine reservoir, it can be subdivided into three zones [28]. The first study site (Site 1) is located 6 km downstream the Misère pre-dam, at the end of the shallow and meandering riverine zone. The reservoir has a width of 125 m and maximum depth of 12 m. Site 2 is located in the lacustrine zone, 7 km downstream of site 1. The reservoir is broader (150 m) and deeper (max. 25 to 30 m, depending on the water level), and it is exposed to wind forcing. Both sites are used for bathing and leisure activities (diving, fishing) during summer months, with peaks of 4,000 individuals per day in the main bathing area [29]. 

At site 1, sample collection was performed directly from a boat and brought back to the beach for on-site filtration. A total of 17 points were sampled at depths of 0, −2, −6 and −10 m (Figure 1B) using a submersible 36 V-booster pump (SQ Deep Well pump, Grundfos).

**Figure 2:**
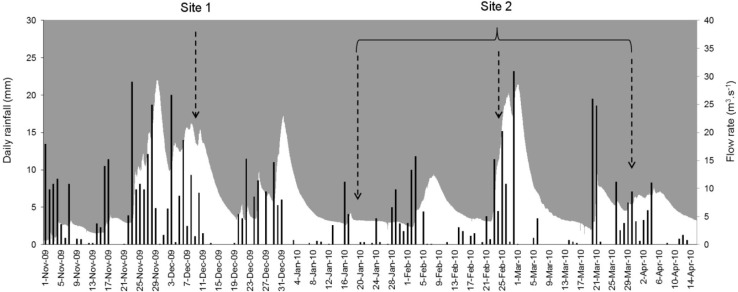
Hydro-climatological conditions during the study period and time of sampling at site 1 and site 2. Daily rainfall (black bars) and flow rates (white area) of the Sûre River (near reservoir inlet) are illustrated from November 2009 to mid-April 2010.

At site 2, 23 to 25 samples were collected at 0, −2, −6, −14 and −24 m using a floating bridge that connects opposite shores. Between 0 and –6 meters, sampling was performed with an electric booster pump (Ironside Garden). Deeper samples (−14 and −24 m) were collected using the submersible booster pump cited above. The cross-section at site 1 was sampled in December 2009 following a prolonged rainfall episode (one-week cumulative rainfall of 57 mm before sampling) (Figure 2). Site 2 was sampled at three successive occasions in January, February and March 2010 (one-week cumulative rainfall of 15, 19 and 19 mm, respectively).

For all sampling points, a volume of 100 L was collected in high-density polyethylene jars preliminarily treated with peracetic acid (Actril Cold Sterilant) and thoroughly rinsed with tap water. Before sampling, jars were further rinsed with reservoir water. For each sample, temperature, pH, conductivity and dissolved oxygen were measured on-site using a portable multi-parameter meter (HQ40d, HACH), and turbidity was assessed with a portable turbidimeter (2100Q, HACH). For enumeration of *E. coli* and total coliforms, 100 mL were transferred to appropriate sterile recipients. Additional aliquots were stored in 2 L-bottles for analysis of total suspended solids (TSS). The remaining volume was used for parasitological analyses. 

### 2.2. Sample Processing

Immediately after sample collection, water was filtered *in situ* using Envirochek HV filter capsules (Pall Corporation) at a flow rate between 2 and 4 L·min^−1^. Filters were kept on ice until arrival at the laboratory and were stored at 4 °C before being processed the following day using the ISO 15553 standard protocol as previously described [27]. In brief, after elution of the capsule filters, samples were concentrated by centrifugation (1100 *g*) followed by immunomagnetic separation (Dynabeads G/C Combo kit, Invitrogen) before immunofluorescence staining with 4’,6’-diamidino-2-phenylindole (DAPI) and fluorescein isothiocyanate (FITC)-conjugated anti-*Cryptosporidium* and anti-*Giardia* monoclonal antibodies (AquaGlo, Waterborne Inc.). Stained (oo)cysts were enumerated by epifluorescence microscopy (DMRB, Leica) and counts were standardized to a volume of 10 L.

Samples for bacteriological analyses (*E. coli* and total coliforms) were kept on ice and protected from sunlight until analysis within 12 h using the Colilert-18 method (IDEXX, UK). For total suspended solids (TSS), membrane filtration was performed (Grade GF/C, Whatman, 1.2 μm pore size). Subsequently, filters were dried at 105 °C during 48 h. Daily rainfall data of the closest meteorological station (near reservoir inlet) were provided by the Administration des Services Techniques de l’Agriculture (ASTA) [30]. Flow measurements of the Sûre River (Figure 2) upstream the Misère pre-dam were obtained from the Hydro-Climatological Observatory of Luxembourg [31].

### 2.3. Reproducibility Assay

In order to quantify sampling and analytical variability in (oo)cyst detection and measurement of all parameters, an additional campaign was performed at site 2. At six points of the cross-section (C and D at 0, −2 and −6 m; Figure 1B), four 100-L samples were collected consecutively and processed for all parameters as described above. Recovery rates of ISO 15553 method were assessed by seeding four replicates (point C, −2 m) on-site with 100 flow cytometry-sorted (oo)cysts of both *Cryptosporidium parvum* and *Giardia duodenalis* (ColorSeed, BTF, North Ryde, Australia) and processed as described above. ColorSeed (oo)cysts are pre-labelled with Texas Red, which enables to distinguish them from naturally-occurring (oo)cysts. Their counts were used for calculation of recovery rates. 

### 2.4. Drifting Buoy Assays

During sample collection at site 1 and site 2 (March campaign), surface and subsurface flow patterns were measured at 0, −2, −6 m using in-house designed drifting buoys. At site 2, additional measurements were made at −14 m. At total of 7 and 11 drifting buoys were released upstream the cross-section at site 1 and site 2, respectively. Their positions were recorded using a hand-held GPS at the time of deployment and recovery. 

### 2.5. Statistical Analyses

Spatial distribution maps were generated by inverse distance weighting (IDW) using ArcMap 9.3 (ESRI, Wemmel, Belgium). For parasites, *E. coli* and turbidity, value classes were defined using twice the mean standard deviation obtained from the reproducibility experiment. Since *Cryptosporidium* oocyst levels were on average more than five times lower than *Giardia* cyst levels (see “Results”) and because of their similar distribution patterns [32], data from both parasites were compiled into one parameter referred to as “parasites”. BioENV analysis based on weighted Spearman rank correlation [33] was performed using PRIMER v.5 to sort out the combination of variables that best explained parasite distribution patterns. Nonparametric Kruskal-Wallis one-way analysis of variance (ANOVA1) followed by Dunn’s *post hoc* test was performed to question significant differences in parasite, *E. coli* and turbidity levels between different sampling depths.

## 3. Results

A total of 113 samples (including the reproducibility experiment) were analysed. Parasite prevalence was high, with 98.2% (111/113) and 91.2% (103/113) of the samples being positive for *Giardia* cysts and *Cryptosporidium* oocysts, respectively. Overall, *Cryptosporidium* oocysts were less abundant (between 0.1 and 5.1 oocysts·10 L^−1^) than *Giardia* cysts (between 0.3 and 34.6 cysts·10 L^−1^) (Table 1). Recovery rates for quantification of (oo)cysts were 49 ± 10% and 32 ± 11% for *Giardia* and *Cryptosporidium*, respectively. In terms of reproducibility, *Giardia* and *Cryptosporidium* counts displayed average coefficients of variation (CV) of 11.5% and 52.8%, resulting in average standard deviations of 1.5 and 0.6 (oo)cysts·10 L^−1^, respectively. Mean CVs for *E. coli* and turbidity levels were 30.1% and 7.2%, respectively (Table A1).

**Figure 3:**
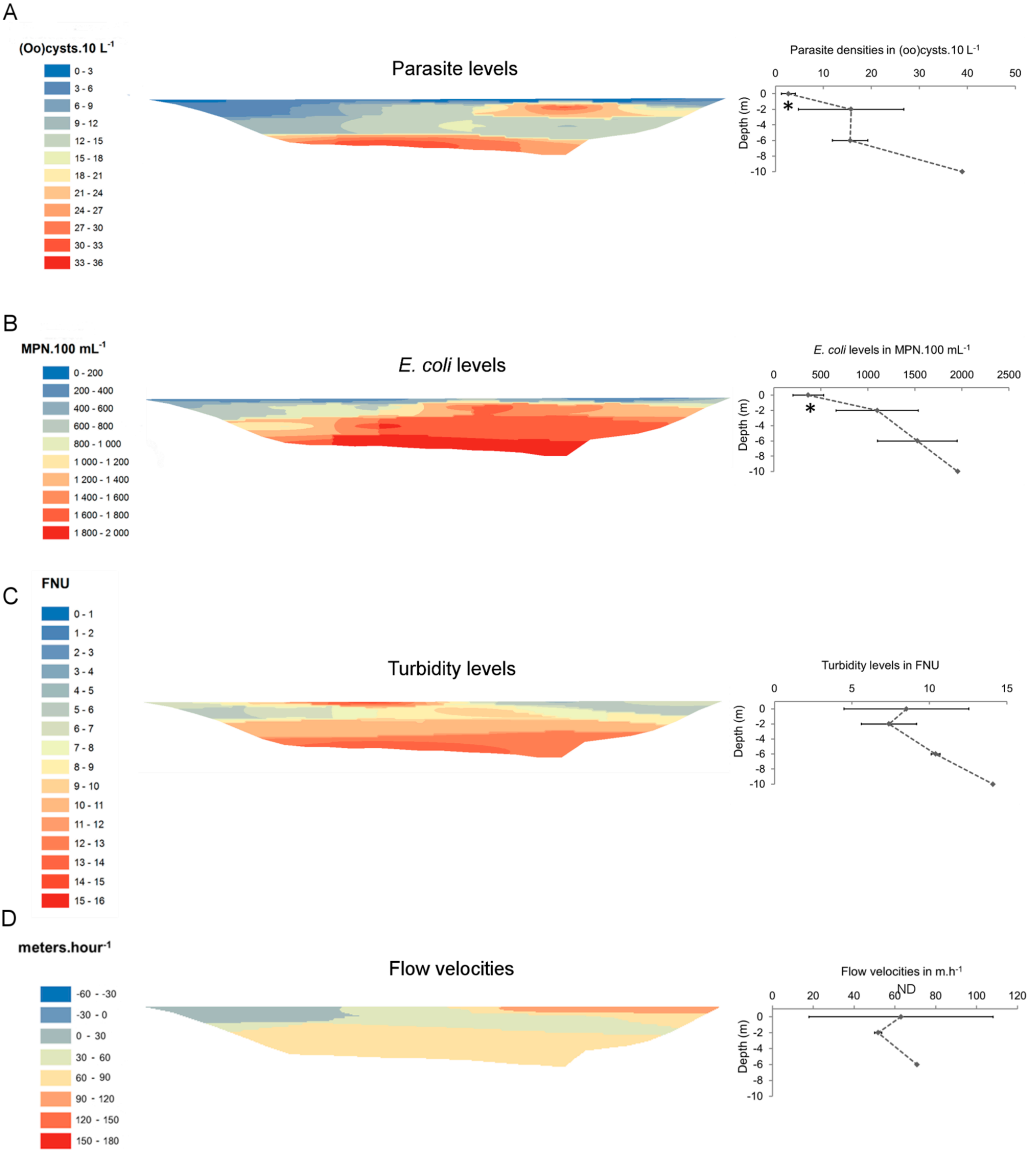
Fine-scale spatial distribution of (**A**) parasites, (**B**) *E. coli*, (**C**) turbidity, and (**D**) flow velocities at site 1 in December 2009. Maps were generated by inverse distance weighting (IDW). Right-hand graphs show the evolution of each parameter along reservoir depth (samples of a same depth were averaged). * significantly (ANOVA1, *p* < 0.05) lower than at −2 and −6 m, FNU: formazin nephelometric units, MPN: most probable number, ND: not determined.

**Figure 4:**
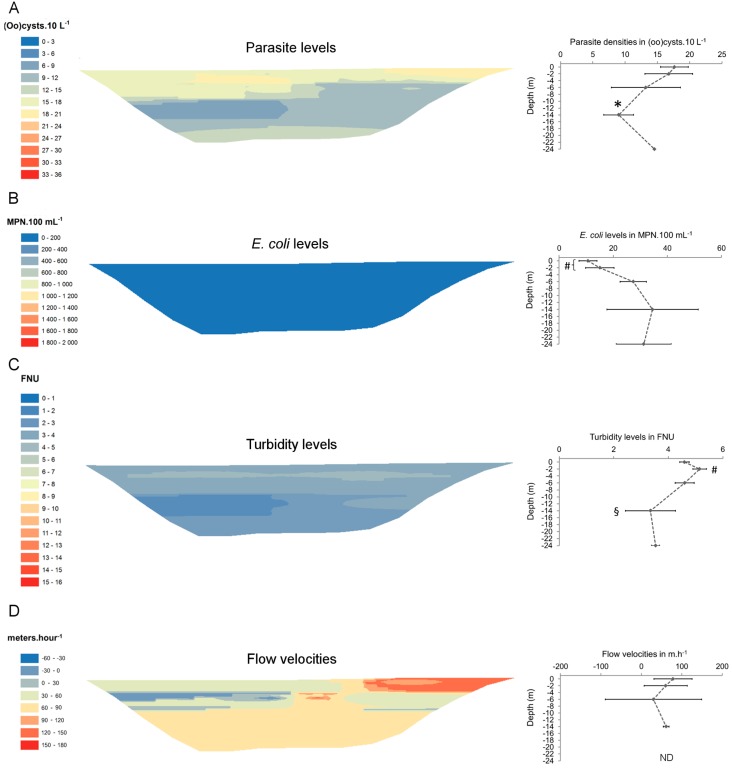
Fine-scale spatial distribution of (**A**) parasites, (**B**) *E. coli*, (**C**) turbidity, and (**D**) flow velocities at site 2 in March 2010. Maps were generated by inverse distance weighting (IDW). Right-hand graphs show the evolution of each parameter along reservoir depth (samples of a same depth are averaged). * significantly (ANOVA1, *p*<0.05) lower than at 0 and -2 metres, ^#^ significantly lower (*E. coli*) or higher (turbidity) than in deeper layers, ^§^ significantly lower than in upper layers, FNU: formazin nephelometric units, MPN: most probable number, ND: not determined.

Parasite spatial distribution was heterogeneous at both sampling sites, and (oo)cyst densities varied significantly with depth. At site 1 (Figure 3), lowest parasite levels were measured at the surface of the reservoir (ANOVA1, *p* < 0.05). Below the surface, they increased locally at −2 m and at the bottom of the reservoir. Levels of *E. coli* were also significantly lower at the surface of the reservoir (ANOVA 1, *p* < 0.05) and increased with depth. Highest turbidity levels (between 10 and 15 FNU) were measured between −6 and −10 meters, but also locally at the surface of the reservoir. At site 2 (March campaign, Figure 4), highest (oo)cyst densities were measured in upper layers of the reservoir (between 0 and −2 m) but dropped below those layers, especially at −14 m (ANOVA 1, *p* < 0.05). Levels of *E. coli* did not exceed 53 MPN·100 mL^−1^, but they were significantly lower between 0 and −2 meters (ANOVA 1, *p* < 0.05). Highest turbidity levels were observed between 0 and −6 m. 

**Table 1:** Microbiological and physical parameters at site 1 and site 2. Values are expressed as average of all sampling points for each cross-section (minimum–maximum), for *Cryptosporidium* and *Giardia*, *E. coli*, total coliforms, turbidity, total suspended solids (TSS) and temperature. Pos.: positive samples, Tot.: total number of samples, FNU: formazin nephelometric units, MPN: most probable number, * 100% positive samples.

Sampling Site/Date		*Cryptosporidium* (oocysts·10L^−1^)		*Giardia* (cysts·10 L^−1^)		*E. coli* * (MPN·100 mL^−1^)	Total Coliforms * (MPN·100mL^−1^)	Turbidity (FNU)	TSS (mg·L^−1^)	Temperature (°C)
Cross-section		Pos./Tot.	Average (min–max)	Pos./Tot.	Average (min–max)	Average (min–max)	Average (min–max)	Average (min–max)	Average (min–max)	Average (min–max)
Site 1	09-Dec-09	14/17	1.9 (0.3–5.1)	17/17	10.6 (1.2–34.6)	992 (256–1956)	3588 (650–9208)	8.9 (4.6–15.7)	4.7 (2.3–8.3)	7.8 (7.1–8.4)
Site 2	19-Jan-10	17/24	0.6 (0.2–2.1)	22/24	2.9 (0.3–6.0)	172 (10–657)	406 (20–1624)	3.4 (2.3–4.4)	1.7 (0.9–2.4)	2.8 (2.3–3.8)
	24-Feb-10	25/25	0.7 (0.1–2.4)	25/25	5.7 (0.4–9.8)	299 (20–2987)	542 (115–3609)	3.3 (1.8–18.2)	2.0 (0.9–9.4)	3.2 (2.2–4.6)
	30-Mar-10	23/23	2.1 (0.3–5.0)	23/23	12.9 (4.0–19.1)	21 (6–53)	106 (55–225)	4.4 (2.7–5.6)	3.0 (1.8–4.0)	8.2 (5.3–10.5)

**Figure A1:**
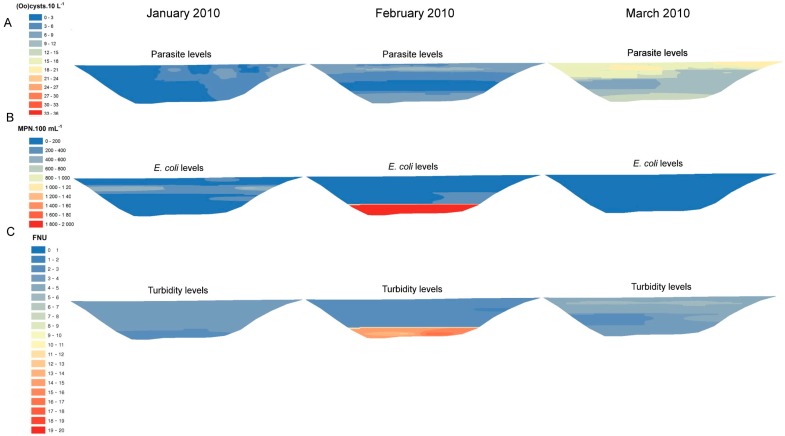
Fine spatial distribution patterns for (**A**) parasites, (**B**) *E. coli* and (**C**) turbidity on three consecutive sampling occasions (January, February and March 2010) at site 2. FNU: formazin nephelometric units, MPN: most probable number.

At the scale of cross-sections, parasite levels increased from January to March, while *E. coli* and turbidity levels did not (Table 1). In February, a local increase in *E. coli* and turbidity levels was observed at the bottom of the reservoir (Figure A1). The combination of variables that best accounted for the spatial distribution of parasites were TSS, *E. coli* and depth at site 1 (Table 2). At site 2, the strongest correlation (*rho* = 0.425), although lower than at site 1, was found for TSS and turbidity.

Surface and subsurface flow velocities varied both vertically and horizontally. At site 1 (Figure 3D), surface flow velocities were higher at the right shore (between 96 and 104 m·h^−1^) than at the left shore (between 9 and 44 m·h^−1^). Intermediate velocities were measured in the middle of the reservoir, between −2 and −6 m. No reverse currents (in upstream direction) were observed. At site 2 (Figure 4D), flow velocities were heterogeneous within the upper layers of the reservoir (between 0 and −6 m) and reverse currents (ranging from 32 to 45 m·h^−1^) were measured at −6 m. Below −6 m, flow patterns were more homogeneous and velocities ranged between 56 and 66 m·h^−1^. Along reservoir width, highest flow velocities (between 111 and 167 m·h^−1^) were observed towards the right shore. 

**Table 2:** Summary of spatial correlations between parasites (regrouping *Cryptosporidium* and *Giardia*) and biotic or abiotic parameters. The combination of variables that best explains the fine-scale spatial distribution of parasites are listed with the *Rho* value, as calculated by BioENV analysis. TSS, total suspended solids

Cross-Section		*Rho* Value	Combination of Variables
Site 1	09-Dec-09	0.784	Depth, *E. coli* and TSS
Site 2	19-Jan-10	0.066	TSS
	24-Feb-10	0.031	Conductivity
	30-Mar-10	0.425	Turbidity, TSS

## 4. Discussion

To date, few data have been gathered on the spatial and temporal scales of *Cryptosporidium* and *Giardia* distribution in lakes and reservoirs [14,27,34,35], but virtually nothing is known about their fine-scale distribution in the water column [16]. Yet, such spatial information is of high relevance for the design of appropriate risk monitoring strategies as well as for the improvement of models predicting (oo)cyst loads in water [36]. In absence of this information, *ad hoc* sampling strategies are likely to underestimate actual parasite densities and misinterpret the associated risk. In this study, we therefore investigated the extent of spatial variability in (oo)cyst densities at fine scale in a drinking water reservoir and provide recommendations for representative sampling within a framework of microbial risk assessment and monitoring.

Direct detection of *Cryptosporidium* and *Giardia* is currently the best way to assess the associated microbial risk in water, but because of their low densities and non-uniform distribution, the protozoan pathogens need to be concentrated from large volumes, resulting in potentially low and variable recovery rates when enumerated by the standard immunofluorescence assay [22,37]. In our study, recovery rates did not significantly differ between *Giardia* and *Cryptosporidium* and were comparable to those already observed in the Upper-Sûre watershed [27]. They were also in the range of those reported elsewhere [38,39,40]. However, based on the coefficients of variation (CV) determined during the reproducibility assay (Table A1), enumeration of *Giardia* was more reproducible than that of *Cryptosporidium*, which is likely attributable to the comparatively lower *Cryptosporidium* oocyst densities found in the reservoir. Other authors have reported very low *Cryptosporidium* densities (<1 oocyst·10 L^−1^) in lakes and reservoirs [24,41]. In the Upper-Sûre watershed, *Giardia* densities were shown to exceed those of *Cryptosporidium* [15,27]. 

High-resolution sampling of reservoir cross-sections revealed a significant heterogeneity in the spatial distribution of parasites. Despite some differences between sampling sites 1 and 2, (oo)cyst densities mainly varied with depth, variations being less pronounced along reservoir width (Figure 3 and Figure 4). At site 1, parasite densities were lowest (<5 (oo)cysts·10L^−1^) at the surface of the reservoir, but increased in deeper layers. Interestingly, this distribution was similar to that of *E. coli*, which, combined to total suspended solids (TSS) did best explain the spatial pattern of parasites (*Rho* = 0.784) (Table 2). A possible explanation for these patterns could be the removal of particles (including *E. coli* and parasites) from surface layers through settling, which is known to occur in the first kilometres of reservoirs [28]. The spatial correlation between parasites, *E. coli* and TSS more generally suggests that biotic and abiotic particles were subject to similar transport dynamics at site 1, probably due to the proximity to the Sûre River inflow (Figure 1). Furthermore, given the successive rainfall episodes that occurred before sampling (Figure 2), large loads of biotic and abiotic runoff particles are assumed to have been introduced into the reservoir and transported towards site 1. 

Field measurements of water movements at the time of sampling support the hypothesis that local hydrodynamics are mainly governed by the Sûre River inflow. Similarly to curved river channels [28], surface flow velocities were highest towards the right shore, while they were lower at the left shore (Figure 3D). Although additional measurements of flow patterns are needed to confirm the hydraulic behaviour of the reservoir at site 1, our results suggest that, at least within the upstream reaches of the reservoir, inflowing runoff particles (including (oo)cysts and *E. coli*) follow a preferential path dictated by valley configuration. This could explain the comparatively low parasite and *E. coli* levels measured near the left shore, where water was also more stagnant. Another hypothesis would be the re-suspension of parasites and *E. coli* from sediments near the right shore [42]. The fine-scale spatial heterogeneity observed at site 1 has major implications for microbial risk monitoring since water samples are generally more readily collected from the left shore (bathing and recreational site). Following that strategy, parasite densities would have been underestimated, whereas a same sample taken in deeper layers (between −2 and −10 m) near the opposite shore would have provided a more representative picture of the actual and potentially highest parasite load.

At site 2, parasite levels also varied significantly with depth, although their horizontal distribution was more uniform than at upstream site 1. As shown in Figure 4A, surface and near-surface layers (0 to −2 m) contained the highest amounts of (oo)cysts and the variability along reservoir width was limited. Parasite densities dropped below −6 m, but increased at the bottom of the reservoir (−24 m), almost equalling those in the upper layers. The latter trend was already observed one month earlier for the same cross-section, with an even more pronounced near-bottom increase in *E. coli* and turbidity levels (Figure A1). This phenomenon could result from the re-suspension of settled material (including (oo)cysts) [42,43,44], as hypothesized for site 1. Reservoir sediments at site 2 can contain up to 2 (oo)cysts·g^−1^, with a large majority of *Giardia* cysts [32]. This amount is not negligible given the load (4 oocysts·g^−1^) reported from polluted sediments [45]. Although it is unknown to what extent re-suspension processes occur in the Upper-Sûre reservoir, it cannot be ruled out that sediments can act as a source of (oo)cysts in the water column, which would consequently require further attention, especially regarding their viability and infectivity. 

Water movements at site 2 turned out to be more complex, as notably illustrated by the occurrence of reverse currents at −6 m (Figure 4D). This part of the reservoir is more exposed to wind forcing than site 1. In addition, the inflow of a tributary that drains a 17 km^2^-large rural catchment (Figure 1) mixes with the reservoir’s mainstream ahead of the cross-section. As a consequence, local hydrodynamics are expected to have a significant impact on the fine-scale spatial distribution of parasites and other suspended particles. Upper layers (0 to −6 m) displayed more heterogeneous flow patterns than deeper layers and also contained highest parasite loads (Figure A1), suggesting that the transport of (oo)cysts is mainly restricted to that part of the water column. Based on our results, the most representative assessment of parasite densities would be obtained for a sample taken from a median point between opposite shores, preferably at −2 m.

In contrast to upstream site 1, we did not observe any spatial link between parasites and *E. coli* at site 2 (Table 2). Absence of consistent correlations between indicators of faecal pollution and pathogens such as *Cryptosporidium* and *Giardia* can result from differences in their respective sources and survival rates in water and sediments [46,47] as well as from different transport dynamics across water bodies. Brookes *et al.* [16] showed that *Cryptosporidium* and faecal indicator bacteria (*Clostridium perfringens* and enterococci) were associated with suspended particles of different size ranges, which in turn would influence their respective settling velocities. Mixing and possible re-suspension processes at site 2 might further complicate any spatial correlation between parasites and *E. coli*. 

Rainfall is considered to be a driving factor for the occurrence of high *Cryptosporidium* and *Giardia* densities in surface waters [48,49] and has a significant role in their distribution dynamics in the Upper-Sûre reservoir [27]. At downstream site 2, highest parasite densities were observed in March (Figure A1). Interestingly, sampling was performed several days after an intense rainfall episode (Figure 2). The generated runoff (or part of it) may have progressively been transported from reservoir inlet towards sampling site 2. Indeed, a similar timing (~10 days) between rainfall onset and detection of parasite peak densities was recorded during the same season at the nearby drinking water treatment plant (DWTP) inlet [27]. In contrast, any rainfall-driven runoff might not have reached site 2 at the time of sampling in January and in February, most likely because of lower rainfall intensities and different timings between rainfall onset and sampling. In addition to the described heterogeneous spatial patterns, this seasonal variability in parasite densities highlights the complex interplay between rainfall and reservoir hydrodynamics. It should be noted that sample collection was performed in absence of thermal stratification. Also, any interaction with protozoan or metazoan grazers can be excluded at that period of the year [17]. As a result, local hydrodynamics and settling/re-suspension processes are likely the main forces governing parasite spatial distribution patterns in the reservoir during the prevalence period.

Because of its relatively short distance from the DWTP inlet (Figure 1), site 2 would be suitable for routine monitoring of parasites and hence provide an early warning to the DWTP. However, given the fact that standard immunofluorescence-based enumeration of *Cryptosporidium* and *Giardia* currently takes more than 24 h, more rapid and cost-affordable methods are needed [50]. Molecular typing as well as on-chip platforms hold promise to reduce sample-to-data-reporting time [51,52,53]. Also, integration of information on oocyst density, infectivity and genotype into one sample as well as simultaneous concentration of parasites and other pathogens (such as viruses) will improve microbial risk assessment of surface waters [25,54,55]. 

## 5. Conclusions

The present study provides unique data on the spatial distribution of pathogenic protozoa and *E. coli* in a drinking water supply. Results highlight the need for the acquisition of minimal knowledge on parasite spatial distribution within the water column in order to avoid misinterpretation of the associated microbial risk through non-representative sampling or the use of faecal indicators such as *E. coli*. Future studies should focus on the hydraulic modelling of the Upper-Sûre reservoir in order to better understand the role of hydrodynamics on (oo)cyst transport [35,56]. Combined with rapid analytical tools, preliminary selection of the most representative locations for parasite monitoring is expected to save significant costs and prevent potentially important public health issues. 

## Appendix

**Table A1:** Analytical reproducibility for microbiological and physical parameters measured at site 2. Six points (C and D at 0, −2 and −6 m) were individually sampled four times consecutively and analysed for all parameters. Values are expressed as average ± standard deviation (n = 4 replicates). Coefficients of variation (CV, expressed in %) are indicated in brackets. FNU: formazin nephelometric units, MPN: most probable number, TSS: total suspended solids. ***** averaged on the 6 sampling points.

Variable	Sampling Point						Mean Standard Deviation (Mean CV) *
	C0	C2	C6	D0	D2	D6	
*Cryptosporidium* (oocysts·10 L^−1^)	0.3 ± 0.2 (54%)	0.4 ± 0.1 (17%)	0.2 ± 0.1 (40%)	0.4 ± 0.4 (89%)	5.9 ± 0.8 (14%)	2.1 ± 2.2 (104%)	0.6 (52.8%)
*Giardia* (cysts·10 L^−1^)	11.5 ± 1.2 (11%)	14.6 ± 1.8 (12%)	12.8 ± 1.8 (14%)	10.0 ± 0.9 (9%)	14.3 ± 1.6 (11%)	14.3 ± 1.7 (12%)	1.5 (11.5%)
*E. coli* (MPN·100 mL^−1^)	17.0 ± 4.5 (26%)	16.4 ± 2.7 (17%)	14.9 ± 2.8 (19%)	17.3 ± 4.2 (24%)	18.9 ± 6.7 (35%)	15.4 ± 9.1 (59%)	5.0 (30.1%)
Total coliforms (MPN·100 mL^−1^)	41.0 ± 8.8 (22%)	38.3 ± 6.8 (18%)	33.8 ± 6.6 (20%)	100.1 ± 92.7 (93%)	42.9 ± 13.5 (31%)	37.9 ± 12.3 (32%)	23.4 (35.9%)
Turbidity (FNU)	3.4 ± 0.3 (8.4%)	3.0 ± 0.2 (5.5%)	3.1 ± 0.2 (7.0%)	3.1 ± 0.1 (4.8%)	3.1 ± 0.1 (3.6%)	3.3 ± 0.5 (14.1%)	0.2 (7.2%)
TSS (mg·L^−1^)	30.7 ± 0.3 (0.9%)	30.6 ± 0.3 (0.9%)	30.6 ± 0.3 (1.1%)	34.4 ± 6.7 (19.4%)	41.8 ± 3.2 (7.7%)	36.9 ± 7.3 (19.8%)	3.0 (8.3%)
Temperature (°C)	3.9 ± 0.2 (5.5%)	4.4 ± 0.1 (3.2%)	4.4 ± 0.1 (2.9%)	4.0 ± 0.2 (4.1%)	4.2 ± 0.2 (4.5%)	4.5 ± 0.1 (2.1%)	0.2 (3.7%)
pH	6.8 ± 0.1 (2.1%)	6.7 ± 0.6 (8.8%)	7.0 ± 0.1 (1.2%)	6.8 ± 0.1 (2.1%)	6.8 ± 0.3 (5.1%)	7.1 ± 0.4 (5.1%)	0.3 (4.0%)
Conductivity (µS·cm^−1^)	154.3 ± 0.7 (0.5%)	153.5 ± 0.2 (0.1%)	153.2 ± 0.7 (0.4%)	154.6 ± 0.7 (0.5%)	153.5 ± 0.7 (0.5%)	153.4 ± 0.4 (0.2%)	0.6 (0.4%)
Dissolved oxygen (mg·L^−1^)	9.6 ± 0.2 (1.9%)	9.1 ± 0.2 (2.0%)	9.3 ± 0.2 (1.9%)	9.5 ± 0.5 (5.3%)	9.0 ± 0.1 (1.7%)	9.6 ± 0.2 (1.7%)	0.2 (2.4%)

## References

[1] White W.R. (2010). World Water: Resources, Usage and the Role of Man-Made Reservoirs.

[2] Brookes J.D., Antenucci J., Hipsey M., Burch M.D., Ashbolt N.J., Ferguson C. (2004). Fate and transport of pathogens in lakes and reservoirs. Environ. Int..

[3] Rose J.B., Gerba C.P., Jakubowski W. (1991). Survey of potable water supplies for *Cryptosporidium* and *Giardia*. Environ. Sci. Technol..

[4] Onichandran S., Kumar T., Lim Y.A., Sawangjaroen N., Andiappan H., Salibay C.C., Chye T.T., Ithoi I., Dungca J.Z., Sulaiman W.Y. (2013). Waterborne parasites and physico-chemical assessment of selected lakes in Malaysia. Parasitol. Res..

[5] Baldursson S., Karanis P. (2011). Waterborne transmission of protozoan parasites: Review of worldwide outbreaks—An update 2004–2010. Water Res..

[6] Mackenzie W.R., Hoxie N.J., Proctor M.E., Gradus M.S., Blair K.A., Peterson D.E., Kazmierczak J.J., Addiss D.G., Fox K.R., Rose J.B. (1994). A massive outbreak in Milwaukee of *Cryptosporidium* infection transmitted through the public water supply. New Eng. J. Med..

[7] Nygard K., Schimmer B., Sobstad O., Walde A., Tveit I., Langeland N., Hausken T., Aavitsland P. (2006). A large community outbreak of waterborne giardiasis-delayed detection in a non-endemic urban area. BMC Public Health.

[8] Widerstrom M., Schonning C., Lilja M., Lebbad M., Ljung T., Allestam G., Ferm M., Bjorkholm B., Hansen A., Hiltula J.I. (2014). Large outbreak of *Cryptosporidium hominis* infection transmitted through the public water supply, Sweden. Emerg. Infect. Dis..

[9] World Health Organisation (WHO) Water Safety Plan Manual (WSP Manual): Step-by-step Risk Management for Drinking-water Suppliers. http://www.who.int/water_sanitation_health/publication_9789241562638/en/.

[10] Dreelin E.A., Ives R.L., Molloy S., Rose J.B. (2014). *Cryptosporidium* and *Giardia* in surface water: A case study from Michigan, USA to inform management of rural surface water systems. Int. J. Environ. Res. Public Health.

[11] Ong C., Moorehead W., Ross A., Isaac-Renton J. (1996). Studies of *Giardia* spp. and *Cryptosporidium* spp. in two adjacent watersheds. Appl. Environ. Microbiol..

[12] Wilkes G., Ruecker N.J., Neumann N.F., Gannon V.P.J., Jokinen C., Sunohara M., Topp E., Pintar K.D.M., Edge T.A., Lapen D.R. (2013). Spatiotemporal analysis of *Cryptosporidium* species/genotypes and relationships with other zoonotic pathogens in surface water from mixed-use watersheds. Appl. Environ. Microbiol..

[13] Dorner S.M., Anderson W.B., Gaulin T., Candon H.L., Slawson R.M., Payment P., Huck P.M. (2007). Pathogen and indicator variability in a heavily impacted watershed. J. Water Health.

[14] Keeley A., Faulkner B.R. (2008). Influence of land use and watershed characteristics on protozoa contamination in a potential drinking water resources reservoir. Water Res..

[15] Helmi K., Skraber S., Burnet J.B., Leblanc L., Hoffmann L., Cauchie H.M. (2011). Two-year monitoring of *Cryptosporidium parvum* and *Giardia lamblia* occurrence in a recreational and drinking water reservoir using standard microscopic and molecular biology techniques. Environ. Monit. Assess..

[16] Brookes J.D., Hipsey M.R., Burch M.D., Regel R.H., Linden L.G., Ferguson C.M., Antenucci J.P. (2005). Relative value of surrogate indicators for detecting pathogens in lakes and reservoirs. Environ. Sci. Technol..

[17] Jacquet V., Lair N., Hoffmann L., Cauchie H.M. (2005). Spatio-temporal patterns of protozoan communities in a meso-eutrophic reservoir (Esch-sur-Sûre, Luxembourg). Hydrobiologia.

[18] Douglas-Helders G.M., O'Brien D.P., McCorkell B.E., Zilberg D., Gross A., Carson J., Nowak B.F. (2003). Temporal and spatial distribution of paramoebae in the water column—A pilot study. J. Fish. Dis..

[19] Bettarel Y., Amblard C., Sime-Ngando T., Carrias J.F., Sargos D., Garabétian F., Lavandier P. (2003). Viral lysis, flagellate grazing potential, and bacterial production in Lake Pavin. Microb. Ecol..

[20] Goddard V.J., Baker A.C., Davy J.E., Adams D.G., De Ville M.M., Thackeray S.J., Maberly S.C., Wilson W.H. (2005). Temporal distribution of viruses, bacteria and phytoplankton throughout the water column in a freshwater hypereutrophic lake. Aquat. Microb. Ecol..

[21] De Wever A., Muylaert K., Van Der Gucht K., Pirlot S., Cocquyt C., Descy J.P., Plisnier P.D., Vyverman W. (2005). Bacterial community composition in Lake Tanganyika: Vertical and horizontal heterogeneity. Appl. Environ. Microbiol..

[22] Ongerth J.E., Saeed F.M.A. (2013). Distribution of *Cryptosporidium* oocysts and *Giardia* cysts in water above and below the normal limit of detection. Parasitol. Res..

[23] Xiao L., Alderisio K.A., Jiang J. (2006). Detection of *Cryptosporidium* oocysts in water: effect of the number of samples and analytic replicates on test results. Appl. Environ. Microbiol..

[24] LeChevallier M.W., Di Giovanni G.D., Clancy J.L., Bukhari Z., Bukhari S., Rosen J.S., Sobrinho J., Frey M.M. (2003). Comparison of method 1623 and cell culture-PCR for detection of *Cryptosporidium* spp. in source waters. Appl. Environ. Microbiol..

[25] King B., Fanok S., Phillips R., Swaffer B., Monis P. (2015). Integrated *Cryptosporidium* assay to determine oocyst density, infectivity and genotype for risk assessment of source and reuse water. Appl. Environ. Microbiol..

[26] Wu J., Long S.C., Das D., Dorner S.M. (2011). Are microbial indicators and pathogens correlated? A statistical analysis of 40 years of research. J. Water Health.

[27] Burnet J.B., Penny C., Ogorzaly L., Cauchie H.M. (2014). Spatial and temporal distribution of *Cryptosporidium* and *Giardia* in a drinking water resource: Implications for monitoring and risk assessment. Sci. Total Environ..

[28] Wetzel R.G. (2001). Water movements. Limnology: Lake and River Ecosystems.

[29] CRHS État Des Lieux De La Haute-Sûre (in French). http://www.crhs.eu/index.php?id=8;lang=fr.

[30] Agrometeorological Measurement Network of Luxembourg. www.agrimeteo.lu.

[31] Hydro-Climatological Observatory of Luxembourg. www.hydroclimato.lu.

[32] Burnet J.B. (2012). Spatial and Temporal Dynamics of *Cryptosporidium* and *Giardia* in a Drinking Water Reservoir and its Catchment: The Upper-Sûre Reservoir Complex. PhD Thesis.

[33] Clarke K.R., Warwick R.M. (1998). Quantifying structural redundancy in ecological communities. Oecologia.

[34] Van Breemen L.W.C.A., Ketelaars H., Hoogenboezem W., Medema G. (1998). Storage reservoirs—A first barrier for pathogenic micro-organisms in the Netherlands. Water Sci. Technol..

[35] Gallas-Lindemann C., Sotiriadou I., Plutzer J., Karanis P. (2013). Prevalence and distribution of *Cryptosporidium* and *Giardia* in wastewater and the surface, drinking and ground waters in the Lower Rhine, Germany. Epidemiol. Infect..

[36] Antenucci J.P., Brookes J.D., Hipsey M.R. (2005). A simple model for quantifying *Cryptosporidium* transport, dilution, and potential risk in reservoirs. J. Am. Water Works Ass..

[37] Emelko M.B., Schmidt P.J., Reilly P.M. (2010). Particle and microorganism enumeration data: Enabling quantitative rigor and judicious interpretation. Environ. Sci. Technol..

[38] Ferguson C., Kaucner C., Krogh M., Deere D., Warnecke M. (2004). Comparison of methods for the concentration of *Cryptosporidium* oocysts and *Giardia* cysts from raw waters. Can. J. Microbiol..

[39] US EPA (2005). Method 1623: *Cryptosporidium* and *Giardia* in Water by Filtration/IMS/FA. http://water.epa.gov/scitech/methods/cwa/bioindicators/upload/method_1623.pdf.

[40] Zuckerman U., Tzipori S. (2006). Portable continuous flow centrifugation and method 1623 for monitoring of waterborne protozoa from large volumes of various water matrices. J. Appl. Microbiol..

[41] Edzwald J.K., Kelley M.B. (1998). Control of *Cryptosporidium*: From reservoirs to clarifiers to filters. Water Sci. Technol..

[42] Quilliam R.S., Clements K., Duce C., Cottrill S.B., Malham S.K., Jones D.L. (2011). Spatial variation of waterborne *Escherichia coli*—Implications for routine water quality monitoring. J. Water Health.

[43] Hawley N., Wang X., Brownawell B., Flood R. (1996). Resuspension of bottom sediments in Lake Ontario during the unstratified period, 1992–1993. J. Great Lakes Res..

[44] Chung G.E., Bonbardelli F.A., Shladow S.G. (2009). Sediment resuspension in a shallow lake. Water Resour. Res..

[45] Molloy S.L., Montgomery A.E., Huffman D.E., Rose J.B. (2006). Detection of *Cryptosporidium parvum* oocysts in sediment and biosolids by immunomagnetic separation. Water Environ. Res..

[46] Staley C., Reckhow K.H., Lukasik J., Harwood V.J. (2012). Assessment of sources of human pathogens and faecal contamination in a Florida freshwater lake. Water Res..

[47] Nasser A.M., Zaruk N., Tenenbaum L., Netzan Y. (2003). Comparative survival of *Cryptosporidium*, coxsackievirus A9 and *Escherichia coli* in stream, brackish and sea waters. Water Sci. Tech..

[48] Young I., Smith B.A., Fazil A. (2015). A systematic review and meta-analysis of the effects of extreme weather events and other weather-related variables on *Cryptosporidium* and *Giardia* in fresh surface waters. J. Water Health.

[49] Atherholt T.B., LeChevallier M.W., Norton W.D., Rosen J.S. (1998). Effect of rainfall on *Giardia* and *Crypto*. J. Am. Water Works Ass..

[50] Weintraub J.M. (2006). Improving *Cryptosporidium* testing methods: A public health perspective. J. Water Health.

[51] Aw T.G., Rose J.B. (2012). Detection of pathogens in water: from phylochips to qPCR to pyrosequencing. Curr. Opin. Biotechnol..

[52] Bridle H., Miller B., Desmulliez M.P.Y. (2014). Application of microfluidics in pathogen monitoring: A review. Water Res..

[53] Burnet J.B., Ogorzaly L., Tissier A., Penny C., Cauchie H.M. (2013). Novel quantitative TaqMan real-time PCR assays for detection of *Cryptosporidium* at the genus level and genotyping of major human and cattle-infecting species. J. Appl. Microbiol..

[54] Bonilla J.A., Bonilla T.D., Abdelzaher A.M., Scott T.M., Lukasik J., Solo-Gabriele H.M., Palmer C.J. (2015). Quantification of protozoa and viruses from small water volumes. Int. J. Environ. Res. Public Health.

[55] Hill V.R., Polaczyk A.L., Kahler A.M., Cromeans T.L., Hahn D., Amburgey J.E. (2009). Comparison of hollow-fiber ultrafiltration to the usepa viradel technique and usepa method 1623. J. Environ. Qual..

[56] Hoyer A.B., Schladow S.G., Rueda F.J. (2015). A hydrodynamics-based approach to evaluating the risk of waterborne pathogens entering drinking water intakes in a large, stratified lake. Water Res..

